# Impaired astrocytic synaptic function by peripheral cholesterol metabolite 27-hydroxycholesterol

**DOI:** 10.3389/fncel.2024.1347535

**Published:** 2024-04-08

**Authors:** Fokion Spanos, Gorka Gerenu, Julen Goikolea, María Latorre-Leal, Hugo Balleza-Tapia, Karen Gomez, Laura Álvarez-Jiménez, Antonio Piras, Marta Gómez-Galán, André Fisahn, Angel Cedazo-Minguez, Silvia Maioli, Raúl Loera-Valencia

**Affiliations:** ^1^Department of Neurobiology Care Sciences and Society, Division of Neurogeriatrics, Karolinska Institutet, Center for Alzheimer Research, Stockholm, Sweden; ^2^Department of Physiology, Biogipuzkoa Health Research Institute - Ikerbasque Basque foundation for Science and University of Basque Country, San Sebastian, Spain; ^3^CIBERNED (Ministry of Economy and Competitiveness, Institute Carlos III), Madrid, Spain; ^4^Anestesiologi Laboratory, Department of Physiology and Pharmacology, Karolinska Institutet, Stockholm, Sweden; ^5^School of Medicine and Health Sciences, Tecnologico de Monterrey, Chihuahua, Mexico

**Keywords:** astrocytes, Alzheimer’s disease, cholesterol metabolism, 27-hydroxycholesterol, 3D co-culture system, synaptic dysfunction, neurospheroid

## Abstract

Astrocytes represent the most abundant cell type in the brain, where they play critical roles in synaptic transmission, cognition, and behavior. Recent discoveries show astrocytes are involved in synaptic dysfunction during Alzheimer’s disease (AD). AD patients have imbalanced cholesterol metabolism, demonstrated by high levels of side-chain oxidized cholesterol known as 27-hydroxycholesterol (27-OH). Evidence from our laboratory has shown that elevated 27-OH can abolish synaptic connectivity during neuromaturation, but its effect on astrocyte function is currently unclear. Our results suggest that elevated 27-OH decreases the astrocyte function *in vivo* in Cyp27Tg, a mouse model of brain oxysterol imbalance. Here, we report a downregulation of glutamate transporters in the hippocampus of CYP27Tg mice together with increased GFAP. GLT-1 downregulation was also observed when WT mice were fed with high-cholesterol diets. To study the relationship between astrocytes and neurons, we have developed a 3D co-culture system that allows all the cell types from mice embryos to differentiate *in vitro*. We report that our 3D co-cultures reproduce the effects of 27-OH observed in 2D neurons and *in vivo*. Moreover, we found novel degenerative effects in astrocytes that do not appear in 2D cultures, together with the downregulation of glutamate transporters GLT-1 and GLAST. We propose that this transporter dysregulation leads to neuronal hyperexcitability and synaptic dysfunction based on the effects of 27-OH on astrocytes. Taken together, these results report a new mechanism linking oxysterol imbalance in the brain and synaptic dysfunction through effects on astrocyte function.

## Background

Alzheimer’s disease (AD) is the main cause of dementia in the aging population and a growing global epidemic (Nussbaum and Ellis, 2003). Several of the main risk factors for developing late-onset AD are genes related to cholesterol metabolism, including cholesterol transport apolipoproteins (Cedazo-Minguez et al., 2001; Cedazo-Minguez and Cowburn, 2001; Rahman et al., 2005; Kivipelto et al., 2008; Cedazo-Minguez et al., 2011; Maioli et al., 2012). At first look, this evidence seemed contradictory since cholesterol is unable to pass the blood–brain barrier; however, extensive research uncovered how cholesterol metabolism in the brain is regulated by an interplay between its metabolites, namely 24S-hydroxycholesterol (24-OH) and 27-hydroxycholesterol (27-OH) (Bjorkhem et al., 2009).

The interplay of 24-OH and 27-OH is crucial for neuronal cholesterol metabolism regulation, neuronal survival, and synaptic function (Bjorkhem, 2006). 27 − OH represents the predominant cholesterol metabolite in plasma, originating from the enzymatic activity of CYP27A1 (Meaney et al., 2002). This catalytic function is pervasive across various tissues, all capable of 27-hydroxylation (Cali and Russell, 1991). Subsequently, this metabolite is released into the systemic circulation, and its concentration correlates directly with plasma cholesterol levels (Babiker et al., 2005). Due to its ability to traverse lipophilic membranes, 27 − OH undergoes flux across the blood–brain barrier (BBB), entering the brain from the systemic circulation (Meaney et al., 2002). Nevertheless, under non-pathological conditions, the cerebral levels of 27 − OH are maintained at minimal levels owing to a highly efficient metabolic process (Goldstein et al., 2006).

Findings from several studies suggest that increased 27-OH levels in the brain might accelerate neurodegeneration. For example, the activity-regulated cytoskeleton-associated protein (ARC), which is involved in memory formation, is downregulated in response to 27-OH, consistent with the low levels of this protein in AD patients (Mateos et al., 2009). Previous evidence from our group has suggested the inhibitory role of oxysterols in neuromaturation and cognition (Heverin et al., 2015). From behavioral tests in Cyp27Tg, we have reported learning and behavioral deficits induced by high 27-OH levels (Heverin et al., 2015; Ma et al., 2015; Ismail et al., 2017). We recently described in young mice that high levels of 27-OH downregulate the postsynaptic protein (PSD-95) and synaptosome-associated protein 25 (SNAP-25) levels in the hippocampus of Cyp27Tg mice together with morphological alterations in neuronal structure and dendritic spines density in CA1 pyramidal neurons (Merino-Serrais et al., 2019; Loera-Valencia et al., 2021b). While we previously found aberrant long-term potentiation (LTP) in Cyp27Tg mice, neuronal glutamate receptor levels were not altered (Loera-Valencia et al., 2021b), which suggests that other synaptic mechanisms can participate in the 27-OH mediated damage to brain function.

Astrocytes have a crucial role in the efficiency of synaptic transmission in the brain, where their activity directly impacts the cognitive performance and behavior of animal models (Windrem et al., 2014). An important role of astrocytes in synaptic function comes from the recapture of glutamate from the synaptic cleft for its conversion to L-glutamine, which is then shuttled back to the presynaptic neuron to generate new glutamate for neurotransmission (Di Castro et al., 2011; Panatier et al., 2011). AD brains have impaired glutamate transporter function, lower levels of the protein glutamate transporter-1 (GLT-1) and increased levels of glial fibrillar associated protein (GFAP) (Masliah et al., 1996; Rothstein et al., 1996; Kirvell et al., 2006; Garcia-Esparcia et al., 2018); however, the mechanisms of how AD leads to astrocyte dysfunction in the brain are not yet fully understood. Moreover, astrocytes are an important source of cholesterol for neurons, and neurodegeneration is induced by the loss of cholesterol delivery from astrocytes in LXR mutant mice (Andersson et al., 2005).

27-OH modifies gene expression through LXR activation in the brain and has also been shown to be a stress signal for both neurons and astrocytes (Cedazo-Minguez et al., 2011; Heverin et al., 2015; Barone et al., 2019). Therefore, oxysterols such as 27-OH have the potential to interfere with normal astrocyte function, including signaling with neurons. We recently reported that high levels of 27-OH could activate astrocytes and induce sterile inflammation in mouse brains through the retinoid receptor X gamma (RxRγ) (Loera-Valencia et al., 2021a); yet, detailed information about the effect of high 27-OH on astrocyte synaptic function is lacking. Here, we report that high levels of 27-OH induce astrocyte activation *in vivo* and *in vitro*, together with a downregulation in glutamate transporters GLT-1 and GLAST. When co-cultured in three dimensions (3D), neurons and astrocytes treated with high 27-OH levels display glutamate transporter downregulation and electrical hyperexcitability, as expected from the dysfunction of synaptic glutamate recapture. To the best of our knowledge, this is the first description of a synaptic dysfunction mechanism involving 27-OH and astrocytic glutamate recapture function in the brain.

## Methods

### Cyp27Tg mice

We used a transgenic mouse model overexpressing the enzyme Cyp27A1 named Cyp27Tg (Meir et al., 2002). Since Cyp27A1 converts cholesterol to 27-OH (Bavner et al., 2010), Cyp27Tg mice have 6 times higher levels of 27-OH than wild-type mice (WT) in serum with normal brain cholesterol amounts (Alberdi et al., 2013; Ismail et al., 2017). Age-matched WT mice served as controls. The mice were fed normal chow, water was provided *ad libitum,* housing maintained a 12-h light/dark cycle. Only 2-month-old males were used.

### High-Fat/high-cholesterol diet mice

Mice aged 5 to 6 weeks (C57BL/6 strain) were procured from B&K (Sollentuna, Sweden). The mice were categorized according to their diets into two groups: one receiving a normal chow diet (ND) and the other a high-fat diet (HFD), comprising 21% fat and 0.15% cholesterol (R638, Lactamine, Sweden) for 9 months as previously reported by our group (Loera-Valencia et al., 2021a).

For immunoblotting analyses HFD and Cyp27Tg animals were sacrificed by isoflurane sedation following decapitation. The brains were dissected and immediately frozen on dry ice and stored at −80°C until processing. A whole right hippocampus or cortex per animal was included in the Western blot analyses.

### Cell cultures and treatments

#### Two-dimensional (2D) primary neuronal and astrocyte cultures

We dissected cortico-hippocampal tissues from 16-day-old mouse embryos. For astrocytes, single cell suspension from dissociated tissues was cultured in Dulbecco’s modified Eagles medium (DMEM/F12) supplemented with 10% inactivated fetal bovine serum (FBS) (Life Technologies, Sweden) in 75 cm2 plastic culture flasks (Corning, NY, United States). Cultures were incubated at 37°C, 95% air/5% CO_2_, and culture media were replaced biweekly. Inactivated astrocytes dominated cultures at 10–14 days (Cedazo-Minguez et al., 2001). For neuronal cultures, single-cell suspension from dissociated tissues was cultured in complete neurobasal media (GIBCO) following the manufacturer’s instructions and as previously described by our group (Mateos et al., 2009).

#### 3D Primary co-cultures

Single cells were isolated from acutely dissected cortex-hippocampus tissue from E19 mouse embryos using mild trypsinization and mechanical dissociation. After this, we estimated the suspension cell concentration, controlling for viability using trypan blue (Sigma). Next, we mixed 80,000 cells with liquid cold Matrigel (hESC-Qualified, Thermo Fischer) to form equal volume spheroids at constant cell densities for culture in either glass-bottom plates for immunohistochemistry or plastic 6-well plates for qPCR. Defined serum-free media was used with B-27 and N2 Supplements in complete neurobasal media (GIBCO). A single spheroid was derived from one embryo (Figure 3A). We obtained several embryos from each mother, and at least two mothers were used for 3D co-culture experiments. We treat each embryo as a separate individual.

#### 2D and 3D culture treatments

The 27-OH was obtained from Steraloids (Newport, Rhode Island, United States). Treatments were done at 1DIV with either 27-OH or DMSO vehicle at a concentration of 1 μM for 24 h. The cultures were then allowed to continue until 10 DIV, when they were processed for immunostaining or qPCR.

#### 27-OH induced media experiments

2D primary astrocyte cultures were treated daily for 2 or 4 consecutive days with 27-OH 1 μM at 1DIV. Then, the media was changed, and the cultures were allowed to reach 10 DIV. At the end of the culture, the induced media was collected and added 1:10 in complete neurobasal media as a treatment for 2D primary neurons at 1DIV. Then, it was allowed to continue until 10 DIV and processed for qPCR.

### RNA extraction and real-time RT-PCR

RNA extraction and real-time PCR were performed as previously described (Cedazo-Minguez et al., 2011). In brief, total RNA was extracted using the RNeasy lipid tissue mini kit from Qiagen (Palo Alto, CA, United States) following the manufacturer’s instructions. 3D spheroids were lysed directly using 250 microliters of the lysis buffer from the kit, and mRNA extraction was done using a column. cDNA was synthesized from mRNA using oligo dT primers and a reverse-transcription reaction according to the manufacturer’s protocol (Life Technologies CA, USA). Real-time PCR amplification assay for target genes was performed with a total volume of 20 μL in each well containing 10 μL of PCR Master Mix (Life Technologies, CA, United States), 2 μL of cDNA corresponding to 10 ng of RNA, and 1 μL of each TaqMan Gene Expression Assays. Taqman probes were used to specifically amplify Psd95 (Dlg4), Gfap, Glt-1 (Slc1a2/EEAT2), Glast (Slc1a3/EEAT1), Arc, LxRβ (Nr1h2), and RxRγ (Rxrg) miRNAs. Relative quantification of the target genes was done using the Livak method, 2–^ΔΔCt^, where ΔΔCt = (*Ct*_target gene_ – *Ct*_GAPDH_)_treated_ –(*Ct*_target gene_ – *Ct*_GAPDH_)_untreated_. After the 2–^ΔΔCt^ calculations of cDNA for every sample in triplicates, the expression was portrayed as a mean ± SEM.

### Immunocytochemistry

#### 2D cultures

Immunocytochemistry was conducted on glial cells derived from primary cultures. The cells were initially seeded at 50% confluence onto coverslips. After 1 DIV, specific treatments were administered, and after the experiment, cells were pre-fixed with 2% paraformaldehyde (PFA) for 2 min, followed by fixation with 4% PFA for 10 min. Subsequently, the cells underwent three washes with phosphate-buffered saline (PBS). All coverslips were subjected to a 30-min blocking step in PBS containing 0.1% Triton-X and 1% bovine serum albumin (BSA). The primary antibodies were GFAP (rabbit anti-mouse, BD Biosciences), and nuclei were stained with DAPI (4, 6-diamidino-2-phenylindole; Sigma) and mounted with ProLong ® Gold antifade reagent for immunofluorescence analysis (Life Technologies, Carlsbad, CA).

#### 3D cultures

After the experiments, the media was removed, and the cells were washed once with PBS before fixation with 2% methanol-free formaldehyde. The samples were blocked with 5% BSA and 0.3% Triton in PBS for 1 h at room temperature. After PBS washes, primary antibodies diluted in blocking solution were added and incubated overnight at room temperature. Primary antibodies used were chicken anti-MAP2 1:500 (Abcam) and mouse anti-GFAP 1:500 (rabbit, BD Biosciences). After washing with PBS once, the samples were incubated with the secondary antibodies diluted in a blocking solution for 2 h at room temperature. Secondary antibodies used were goat anti-chicken 633 1:1000 (ThermoFisher) and donkey anti-mouse 546 (ThermoFisher). DAPI 1 μg/mL was used to stain nuclei. Afterward, the samples were washed once with PBS and were mounted with coverslips using SlowFade™ Gold Antifade (ThermoFisher). Samples were stored at room temperature to prevent solubilization of the Matrigel matrix.

### Imaging and 3D morphometric analysis

Images were taken using a Nikon inverted Ti microscope and a spinning disc confocal system. The fluorescence of DAPI and Alexa 488 was recorded through separate channels. We obtained image stacks of 10–100 image planes with a 20x dry lens (NA, 0.75). No pixels were saturated. After the acquisition, the stacks were analyzed with 3D image processing software—Imaris 9.1 (Bitplane AG, Zurich, Switzerland).

#### Morphometric parameters of neurons and astrocytes in 3D co-cultures

The volume and the length of astrocytes were established blindly by semiautomatic reconstruction using the Filaments tool from Imaris. The *Cells* tool was used to render the volume of the astrocytes and select only the DAPI-positive GFAP signal. The process was automated for batch analysis. Afterward, the statistics of volume and sphericity were extracted. The *Filaments* tool was used to trace the filaments of both neurons and astrocytes. Only neurons with at least one process were counted. The tracing process was done manually to avoid background staining tracing errors. The statistics of the number of the Sholl intersections (10 μm radius of Sholl spheres), filament length (sum), and dendrite straightness were extracted.

### Immunoblotting analysis

Western blot analysis was carried out in hippocampal tissues as described previously. After being transferred to a nitrocellulose membrane (Schleicher & Schuell, Germany), milk-blocked blots were incubated overnight with the primary antibodies. Primary antibodies used were rabbit anti-GLAST 1:500 (NB100-1869, NovusBio), rabbit anti-GLT1 1:1000 (ab41621, Abcam), rabbit anti-actin 1:1000 (A2066, Sigma-Aldrich), mouse anti-GAPDH 1:1000 (ab8245, Abcam), mouse anti-PSD95 1:1000 (ab2723, Abcam), mouse anti-GFAP 1:000 (BD556330, BD Biosciences), rabbit anti-SNAP25 1:1000 (3,926, Cell Signaling), rabbit anti-NeuN 1:1000 (ABN78, Merck Millipore), and mouse anti-MAP2 1:500 (LS-C178331, LSBio) diluted in TBST. Secondary incubation was carried out using anti-rabbit or anti-mouse IRDye infrared IgG antibodies (Li-Cor Biosciences, United States) at a 1:5000 dilution at room temperature. Immunoreactivity was detected using Odyssey CLx Imaging System (Li-Cor Biosciences, USA). The following day, the membranes underwent three washes with TBST before introducing the fluorescent secondary antibody. The secondary antibody was diluted in TBST with Odyssey® Blocking Buffer in TBS at a 1:1 ratio and incubated for 1 h in the dark at room temperature. The secondary antibodies employed included donkey anti-rabbit 680 (926–68,073, LI-COR) and 800 (926–32,213, LI-COR), as well as goat anti-mouse 680 (926–68,072, LI-COR) and 800 (926–32,210, LI-COR). All secondary antibodies were used at a dilution of 1:10,000. The densitometric analyses of bands were done with Image Studio Lite ver. 5.2 (Li-Cor Biosciences, United States).

### Electrophysiology

Patch clamp (whole-cell) recordings were performed with borosilicate glass microelectrodes (4–6 MΩ) from visually identified neurons using IR-DIC microscopy (Scientifica SliceScope, United Kingdom). A potassium-based intracellular solution was used (in mM): 122.5 K-gluconate, 8 KCl, 4 Na_2_ATP, 0.3 NaGTP, 10 HEPES, 0.2 EGTA, and 2 MgCl, set to pH 7.2–7.3 with KOH. Neurons were held at −70 mV for all electrophysiological characterization. Data were recorded with MultiClamp 700B and Axopatch 200B amplifiers (Molecular Devices), sampled at 10 kHz, low-pass filtered at 2 kHz, digitized (Digidata 1440A, Molecular Devices, CA, United Kingdom), and stored on a hard disc using pCLAMP 10.4 software (Molecular Devices).

### Statistical analysis

#### Electrophysiology

Four current steps from 20 to 80 pA with 20 pA increments and 200 ms length in the current clamp configuration were applied to neurons, and the AP fired in each step was quantified. The AP threshold was calculated from a current ramp protocol from 0 to 300 pA (500 ms), and the threshold was determined by the voltage value at which the first AP was fired. Membrane potential was measured in a 60-s length recording in an I = 0 configuration.

EPSCs were measured in voltage clamp configuration and were detected offline using MiniAnalysis software (Synaptosoft, Decatur, GA, United States). Frequency and amplitude were analyzed using Excel software (Microsoft Office) and GraphPad Prism (GraphPad Software, USA) with the results representing average values taken over 1-min periods.

#### Western blots and qPCR

To test the overall effect of the 27-OH treatments, an unpaired Mann–Whitney test was used to compare the means of either densitometry normalized protein values or ΔΔCt values. In all cases, *p* < 0.05 was significant (* < 0.05, ** < 0.01, *** < 0.001, **** < 0.0001).

#### Morphology analysis

Data were analyzed with GraphPad Prism 8.2.1 and plotted either as Mean ± SEM (Sholl intersections per radius) or individual values alongside the mean (all other graphs). The Wilcoxon test was used for paired comparison between two conditions, the two-way ANOVA with Tukey’s correction was used for multiple comparisons (qPCR data), and the Kruskal–Wallis test is used for comparing the distributions (astrocyte volume and sphericity). For dendritic complexity analysis, a two-way ANOVA repeated measures (*P* and *F* values + interaction are shown) followed by a post-hoc multiple Bonferroni test were used to compare values as a function of the distance from the soma. Data values are expressed as mean ± SEM. In all cases, *p* < 0.05 was significant (* < 0.05, ** < 0.01, *** < 0.001, **** < 0.0001).

## Results

### Astrocyte glutamate transporters are reduced in the cortex and hippocampus of CYP27Tg mice and HFD animals

**Figure 1:**
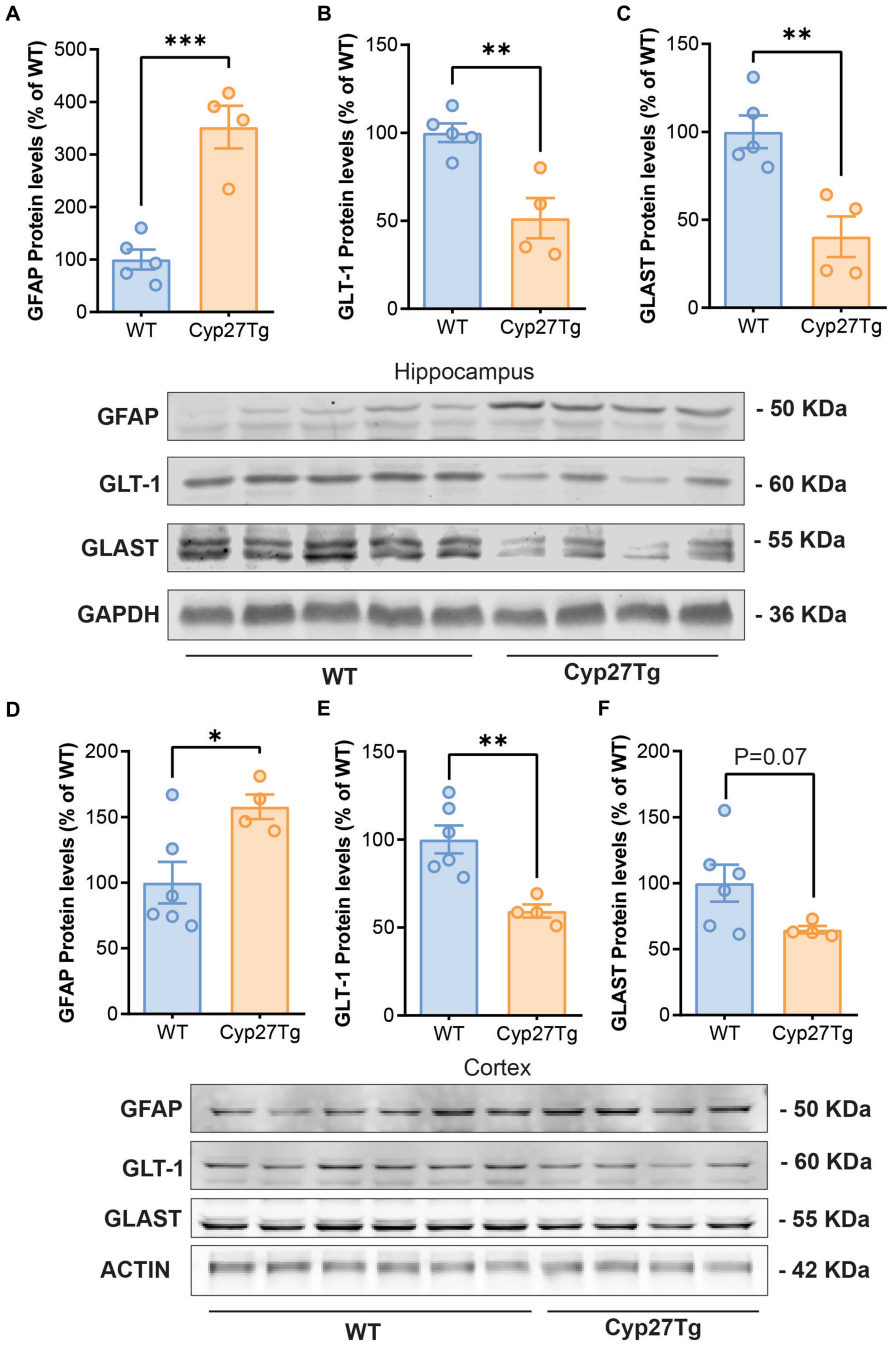
Astrocyte glutamate transporters are reduced in the hippocampus and cortex of CYP27Tg mice. Densitometric analysis of Western blots from Cyp27Tg hippocampus shows increased protein levels of GFAP **(A)** compared to WT animals, with decreased levels of GLT-1 **(B)** and GLAST **(C)**. GFAP protein levels were also increased in Cyp27Tg cortices **(D)**, while GLT-1 was downregulated **(E)**. GLAST levels were not significantly lower than WT animals **(F)**.

Using Western blots, we confirmed an increase in GFAP protein levels in both the cortex and hippocampus of Cyp27Tg mice (Figures 1A,D); Hippocampus: WT group (*n* = 5), mean = 100.0 (±18.96 SEM); Cyp27Tg group (*n* = 4), mean = 352.0 (±40.61 SEM), *p* = 0.0005; Cortex: WT group (n = 6), mean = 100.0 (±15.88 SEM); Cyp27Tg group (*n* = 4), mean = 157.8 (±9.317 SEM), *p* = 0.0260. Following our hypothesis, we quantified astrocyte glutamate transporters GLT-1 and GLAST, and we found that Cyp27Tg mice showed a decrease in both markers in the hippocampus (Figures 1B,C); GLT-1 WT group (*n* = 5), mean = 100.0 (±5.300 SEM); Cyp27Tg group (*n* = 4), mean = 51.43 (±11.44 SEM), *p* = 0.0043; GLAST WT group (*n* = 5), mean = 100.0 (±9.295 SEM); and Cyp27Tg group (*n* = 4), mean = 40.36 (±11.60 SEM), *p* = 0.0048. Only GLT-1 was significantly decreased in cortex (Figures 1E,F); GLT1 WT (*n* = 6), mean = 100.0 (±7.921 SEM), *p* = 0.0043; Cyp27Tg (*n* = 4), mean = 59.35 (±3.765 SEM); GLAST WT (*n* = 6), mean = 100.0 (±13.95 SEM); and Cyp27Tg (*n* = 4), mean = 64.65 (±2.817 SEM), *p* = 0.0791.

**Figure 2:**
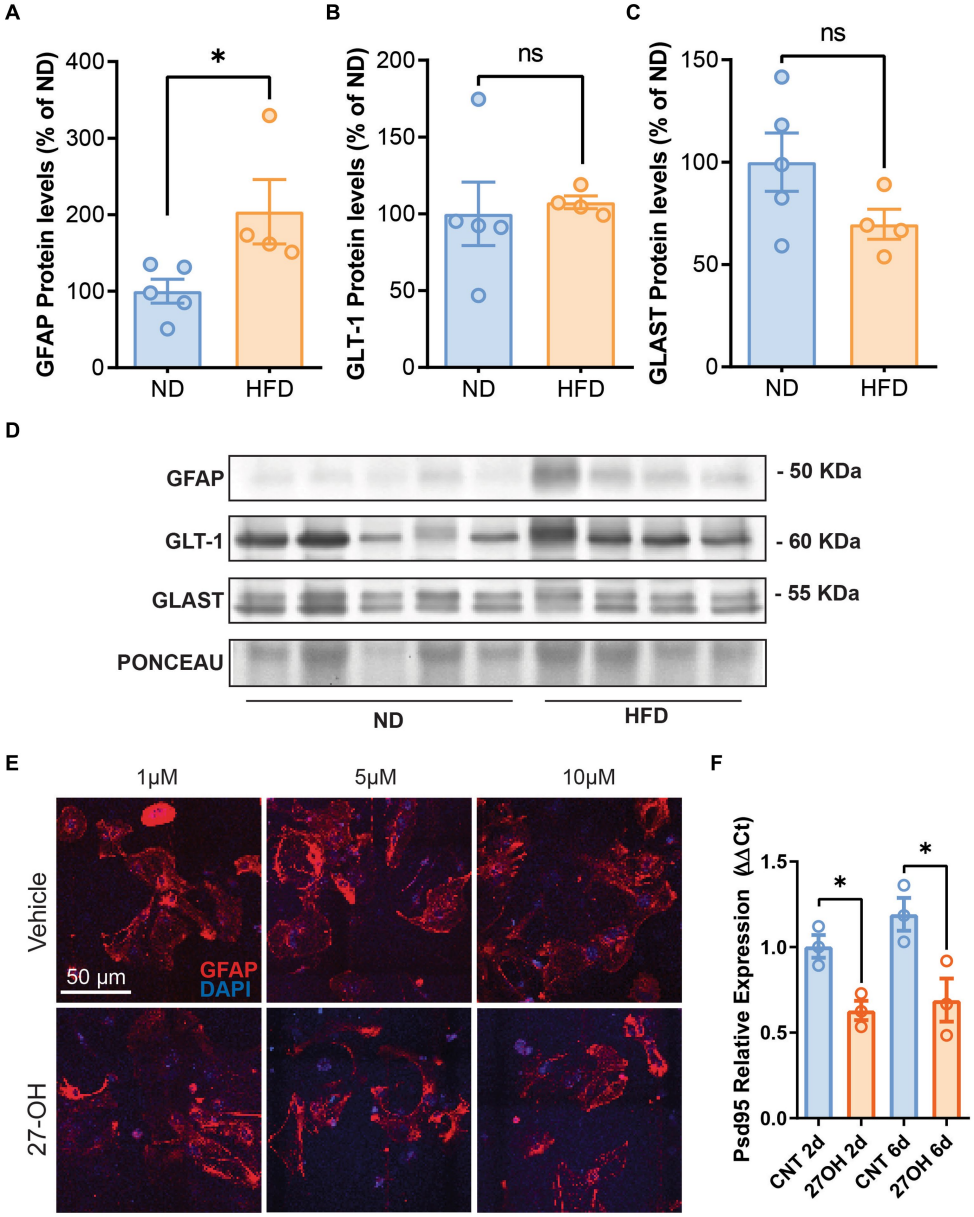
The 27-OH induces astrogliosis in HFD mice but not in 2D astroglial cultures. Densitometric analysis of Western blots from HFD mice hippocampus shows increased protein levels of GFAP **(A)** compared to WT animals but no alterations of GLT-1 **(B)** and GLAST **(C)**. Original image of western blot bands quantified are shown in **(D)** Confocal images from hippocampal 2D primary astrocyte cultures treated with 27-OH 1, 5, and 10 μM showed no signs of stellation **(E)**, but astrocyte-induced media from the 1 μM treatments reduced Psd95 mRNA (Dlg4) expression at 10 DIV in 2D primary neuronal cultures **(F)**.

Previously, we have shown sterile inflammation in HFD mice, which also has increased 27-OH levels in plasma; thus, we interrogated the hippocampus of these animals for astrocyte markers. GFAP was significantly increased compared to WT animals (Figures 2A,D), ND (*n* = 5), mean = 100.0 (±15.58 SEM); HFD (*n* = 4), mean = 203.9 (±42.13 SEM), *p* = 0.0389. However, astrocytic glutamate transporters GLT-1 and GLAST protein levels were not different between groups (Figures 2B–D); GLT-1: ND (*n* = 5), mean = 100.0 (±15.58 SEM); HFD (*n* = 4), mean = 203.9 (±42.13 SEM) *p* = 0.1905. GLAST: ND (*n* = 5), mean = 100.0 (±14.20 SEM); and HFD (*n* = 4), mean = 69.69 (±7.315 SEM), *p* = 0.1238.

### 27-OH-induced media from primary astrocytes decrease Psd95 expression in primary neurons

We treated primary astroglial cultures with 1, 5, and 10 μM concentrations of 27-OH and saw no signs of stellation in the cultures or any morphological alterations in the cells’ characteristic of reactivity (Figure 2E), although we observed an increase in GFAP protein levels with 1 μM 27-OH after 4 and 6 DIV (Supplementary Figure S2 For controls, *n* = 3 per treatments 2DIV, 4DIV, and 6DIV, the mean values were 100.0 ± 2.172, 78.71 ± 14.55, and 163.9 ± 39.69, respectively. 27-OH, n = 3 per treatments 2DIV, 4DIV, and 6DIV; mean values: 109.5 ± 31.01, 158.4 ± 39.18, and 249.7 ± 68.24, respectively). However, when primary neurons in culture were exposed to 27-OH induced media from astrocytes (2 and 4 days of 27-OH 1 μM induction), Psd95 expression decreased at the mRNA level (Figure 2F), which suggested that 27-OH detrimental effects required interaction of neurons and astrocytes. CNT 2d (*n* = 3), mean = 1.004 (±0.06592 SEM); 27OH 2d (*n* = 3), mean = 0.6293 (±0.05616 SEM), *p* = 0.0123; CNT 6d (*n* = 3), mean = 1.192 (±0.09577 SEM); and 27OH 6d (*n* = 3), mean = 0.6901 (±0.1259 SEM), *p* = 0.0338.

### 27-OH induces degeneration and glutamate transporter downregulation in 3D co-cultured astrocytes

**Figure 3:**
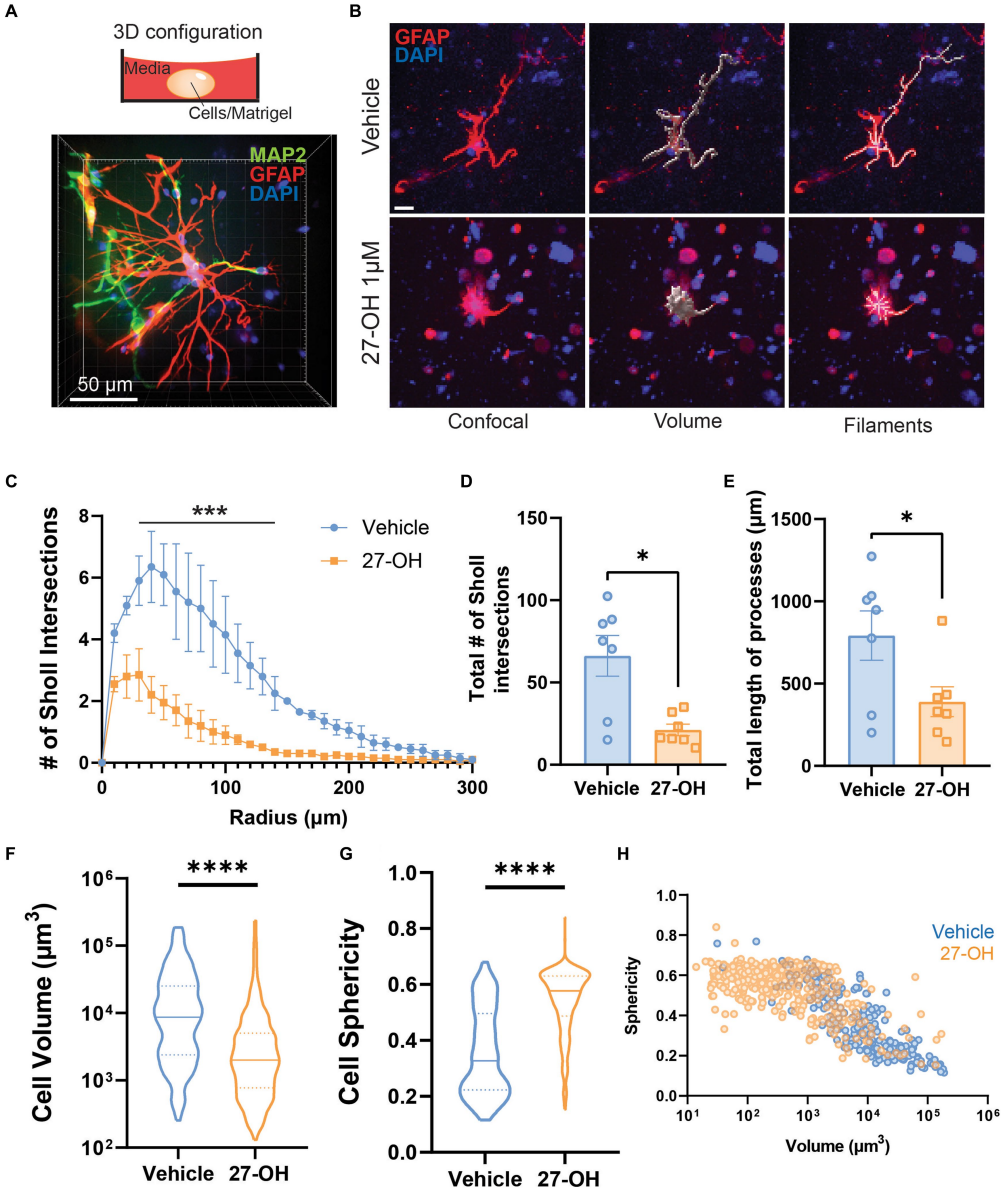
The 27-OH induces degeneration in 3D co-cultured astrocytes. **(A)** 3D configuration of the primary co-cultures from WT mice in a spheroid cultured on dishes for confocal microscopy. Astrocytes are labeled with GFAP (red), neurons with MAP2 (green), and nuclei in blue (DAPI). **(B)** 3D cultured astrocytes treated with vehicle (DMSO, upper panel) or 27-OH 1 μM (lower panel) and labeled with GFAP (red) and DAPI (blue). Z-stacks were processed for morphology analysis using filament tracing and volume reconstructions. The scale bar is 20 μm and applies to all panels. Sholl analysis of 3D-astrocytes shows decreased branching of astrocytes treated with 27-OH **(C,D)** with decreased process length **(E)** compared to controls. **(F)**. Decreased volume of 3D-astrocytes treated with 27-OH and increased sphericity **(G)** as indicators of cell degeneration. **(H)**. Individual astrocytes imaged are represented in the plot to show the shift in overall population in the 3D cultures between the vehicle (blue) and 27-OH (orange) treatments. Total biological replicates *n* = 14 (*p < 0.05, ***p < 0.001, ****p < 0.0001).

We decided to establish a 3D co-culture of primary cells derived from mouse embryos as an experimental model to study the effects of elevated 27-OH levels (Figure 3A). In contrast to 2D primary astroglial cultures, 3D co-cultures showed a clear alteration in astrocyte morphology when treated with 1 μM of 27-OH (Figure 3B). Most of the astrocytes were smaller, with few intersections compared to vehicle controls. We analyzed the volume, sphericity, percentage of reactive astrocytes, number of Sholl intersections, and process length of the astrocytes between the two conditions and found all these parameters reduced in 27-OH treatments (Figures 3C–E). Sholl analysis: Adjusted *p*-values (Holm-Sidak): 0.022834, 0.000411, 0.000875, 0.004487, 0.007428, and 0.014300. Total Sholl: Vehicle (*n* = 7), mean = 66.18 (±12.43 SEM); 27-OH (*n* = 7), mean = 21.19 (±3.504 SEM), *p* = 0.0379; Total length: Vehicle (*n* = 7), mean = 792.4 (±150.0 SEM); 27-OH (*n* = 7), mean = 389.6 (±90.79 SEM), *p* = 0.0404. A significant reduction of cell volume accompanied by an increase in cell sphericity is observed in 27-OH treated 3D cultures (Figures 3F,G). Cell volume: Vehicle (*n* = 213), mean = 21,879 (±2,312 SEM); 27OH (*n* = 438), mean = 7,196 (±1,043 SEM), *p* < 0.0001; Sphericity: Vehicle (*n* = 213), mean = 0.3552 (±0.01062 SEM); 27OH (*n* = 420), mean = 0.5420 (±0.005656 SEM), *p* < 0.0001. The change in 27-OH treated vs. control populations can be observed when individual astrocytes from our experiments are pooled and plotted together as a function of their volume and sphericity (Figure 3H).

**Figure 4:**
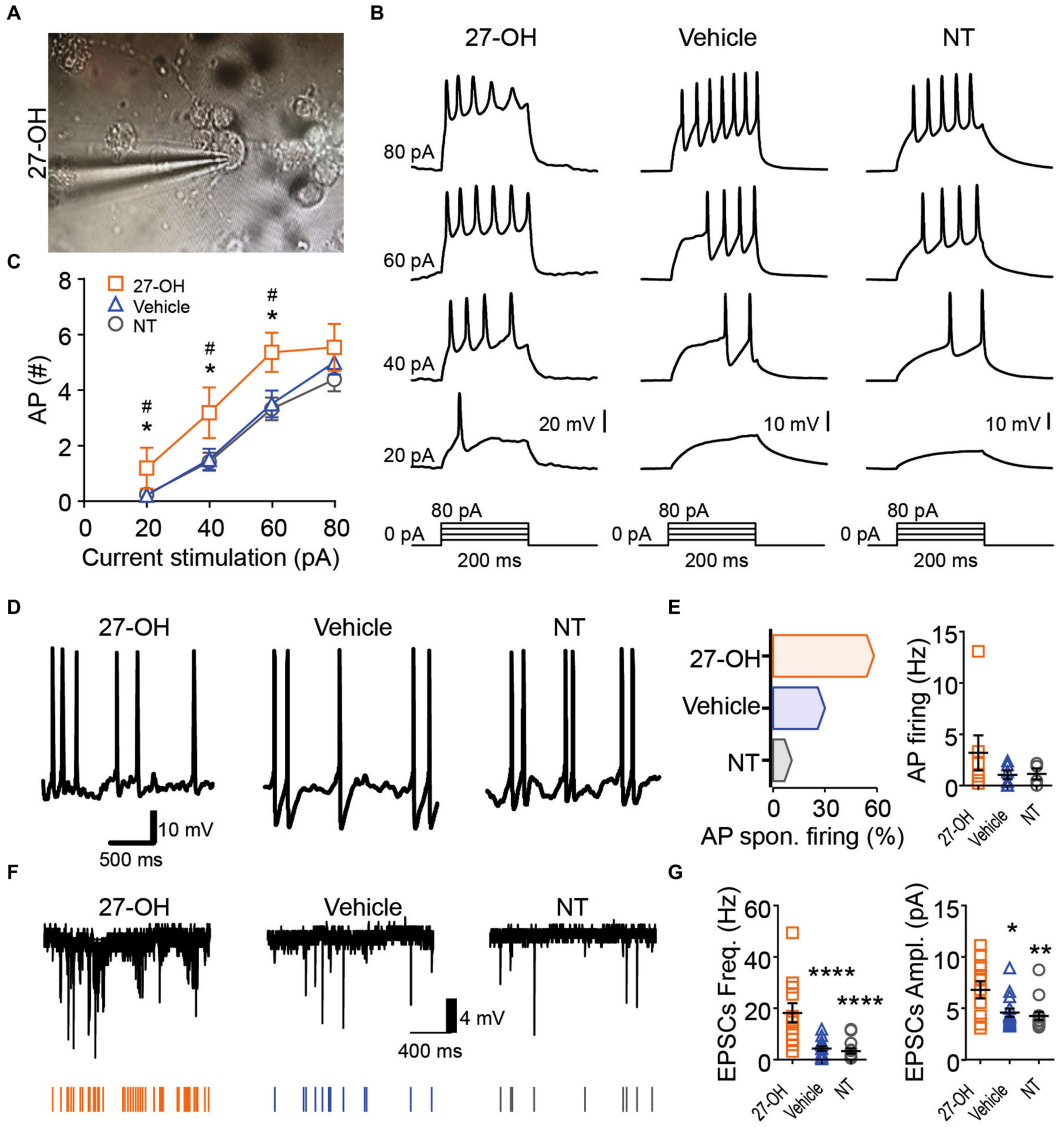
The 27-OH induces hyperexcitability in 3D co-cultured neurons. **(A)** Representative picture showing the morphology from a neuron in 3D cultures treated with 27-OH and the glass pipette used for electrophysiological characterization. **(B)** Representative recording showing the neuron’s response to a step current protocol (bottom), showing the increase in the action potential firing rate of neurons in 3D cultures treated with 27-OH compared to neurons in 3D cultures treated with vehicle (DMSO) and non-treated cultures (NT). **(C)** Relationship between the current stimulation intensity (pA) and the number of AP displayed by neurons in 3D cultures treated with 27-OH (orange squares) or vehicle (blue triangles) and cultures non-treated (gray circles). **(D)** Representative recording of the membrane potential of neurons displaying spontaneous AP firing. **(E)** Percentage of the neurons displaying spontaneous AP firing (left) and quantification of the spontaneous AP firing frequency (right) in 3D cultures treated with 27-OH (orange), DMSO (blue), and neurons in non-treated 3D cultures (NT). **(F)** Representative recordings of the spontaneous excitatory postsynaptic currents (EPSCs) are represented by arrows at the bottom. **(G)** Quantifications showing that neurons in 3D cultures treated with 27-OH display an increase in frequency (left) and amplitude (right) of the EPSCs compared to neurons in DMSO and NT cultures.

**Figure 5:**
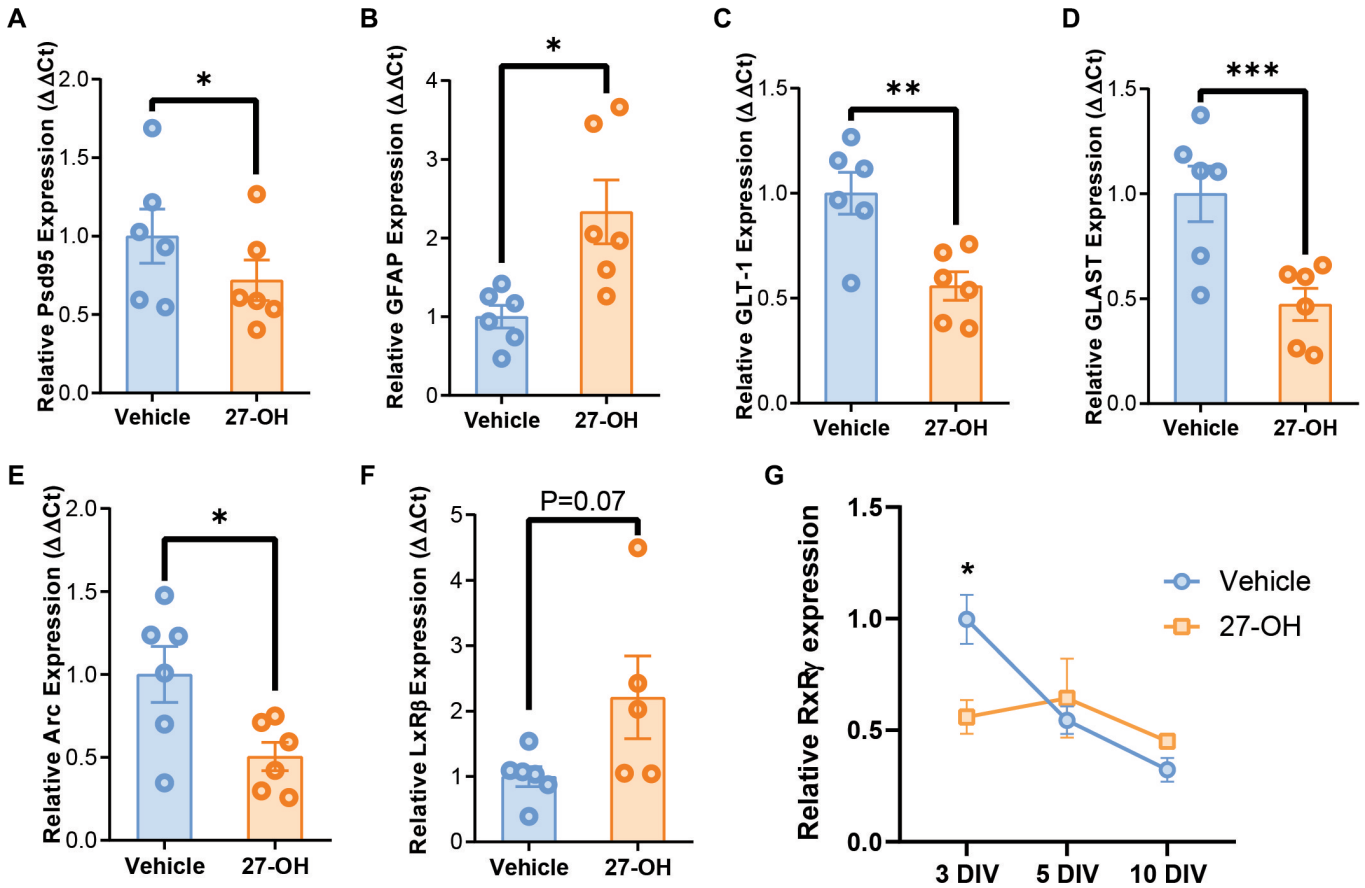
27-OH induces GLT-1 and GLAST downregulation in 3D co-cultures and recapitulates synaptic dysregulation from CYP27Tg mice. mRNA levels of synaptic genes Psd95 (Dlg4) **(A)** and Arc **(E)** are decreased in 3D primary co-cultures treated with 27-OH 1 μM compared to DMSO controls. **(B)** mRNA levels of astrocytic GFAP are increased in cultures treated with 27-OH, while glutamatergic transporters GLT-1 **(C)** and GLAST **(D)** are downregulated compared to vehicle treatments. **(F)** Expression of LxRβ was not significantly increased in 27-OH treated 3D co-cultures. **(G)** RxRγ mRNA levels are decreased at 3DIV in 27-OH treatments compared to its control and do not change during the culture (10DIV), while in controls, it is downregulated throughout the experiment (10DIV). Total biological replicates *n* = 12 (*p < 0.05, ***p < 0.001, ****p < 0.0001).

### 27-OH induces hyperexcitability in 3D co-cultured neurons

In Figure 4, we focused on the impact of 27-OH on excitability in 3D co-cultured neurons. Morphological analysis revealed alterations in neuron structure following treatment with 27-OH (Figure 4A and Supplementary Figure S1). Electrophysiological recordings showed an increase in the action potential firing rate of neurons within 3D cultures treated with 27-OH, compared to both dimethyl sulfoxide (DMSO)-treated and non-treated (NT) cultures, as evidenced by a step current protocol (Figure 4B). The relationship between current stimulation intensity and the number of action potentials further highlighted the heightened excitability induced by the 27-OH treatment (Figure 4C) 20-NT (*n* = 34), mean = 0.2353 (±0.09496 SEM); 20-27OH (*n* = 11), mean = 1.182 (±0.7363 SEM); 20-Vehicle (*n* = 21), mean = 0.1905 (±0.1117 SEM); 40-NT (*n* = 34), mean = 1.412 (±0.3219 SEM); 40-27OH (*n* = 11), mean = 3.182 (±0.9127 SEM); 40-Vehicle (*n* = 21), mean = 1.429 (±0.3689 SEM); 60-NT (*n* = 34), mean = 3.324 (±0.4065 SEM); 60-27OH (*n* = 11), mean = 5.364 (±0.7042 SEM); 60-Vehicle (*n* = 21), mean = 3.333 (±0.4896 SEM); 80-NT (*n* = 34), mean = 4.382 (±0.4243 SEM); 80-27OH (*n* = 11), mean = 5.545 (±0.8460 SEM); 80-Vehicle (*n* = 21), mean = 4.762 (±0.4776 SEM); Vehicle and 27-OH adjusted *p*-values for 20, 40, 60 pA stimulation = 0.0443, 0.0493, 0.0438; NT and 27-OH adjusted p-values for 20, 40, 60 pA stimulation = 0.0443, 0.0458, 0.0413. Additionally, neurons treated with 27-OH exhibited a significant increase in spontaneous action potential (AP) firing compared to DMSO and NT cultures (Figure 4D), quantified as percentage of the neurons displaying spontaneous AP firing and quantification of the spontaneous AP firing frequency (Figure 4E); AP spontaneous firing (%): Non-treated: 10.8, Vehicle: 30, 27-OH, 58.3; AP firing: Non-treated (*n* = 4), mean = 1.138 (±0.5281 SEM); 27OH (*n* = 7), mean = 3.198 (±1.699 SEM); Vehicle (*n* = 6), mean = 1.039 (±0.4239 SEM). Evaluation of membrane potential confirmed this hyperexcitability, with neurons in 3D cultures treated with 27-OH displaying a distinct rise in the frequency and amplitude of spontaneous excitatory postsynaptic currents (EPSCs) compared to control cultures (Figure 4F,G). EPSCs frequency: Non-treated (*n* = 14), mean = 3.383 (±1.025 SEM); 27OH (*n* = 12), mean = 18.23 (±3.773 SEM); Vehicle (*n* = 15), mean = 4.318 (±0.8848 SEM); EPSCs amplitude: Non-treated (*n* = 14), mean = 4.259 (±0.4027 SEM); 27OH (*n* = 12), mean = 6.799 (±0.8256 SEM); Vehicle (*n* = 15), mean = 4.575 (±0.4009 SEM); Adjusted *p*-values: Non-treated vs. 27OH (*p* = 0.0080), Non-treated vs. DMSO (*p* = 0.6741, ns), and 27OH vs. DMSO (*p* = 0.0137). These results collectively underscore the hyper-excitatory influence of 27-OH on 3D co-cultured neurons.

### 27-OH induces GLT-1 and GLAST downregulation in 3D co-cultures and recapitulates synaptic dysregulation from CYP27Tg mice

The expression profile of the primary 3D co-cultures reproduced the behavior of astrocyte markers in the hippocampus, with elevated GFAP mRNA levels (Figure 5B) and downregulated GLT-1 and GLAST levels (Figures 5C,D). GFAP: Vehicle (*n* = 6), mean = 1.000 (±0.1441 SEM); 27-OH (*n* = 6), mean = 2.333 (±0.4053 SEM); GLT-1: Vehicle (*n* = 6), mean = 1.000 (±0.1001 SEM); 27-OH (*n* = 6), mean = 0.5581 (±0.06802 SEM); GLAST: Vehicle (*n* = 6), mean = 1.000 (±0.1316 SEM); 27-OH (*n* = 6), mean = 0.4731 (±0.07641 SEM). In addition, these cultures reproduced downregulation of Psd95 as seen in the induction experiments as well as a decrease in Arc previously reported by our group (Figures 5A,E), Psd95: Vehicle (*n* = 6), mean = 1.000 (±0.1728 SEM); 27-OH (*n* = 6), mean = 0.7186 (±0.1293 SEM); Arc: Vehicle (*n* = 6), mean = 1.000 (±0.1685 SEM); 27-OH (*n* = 6), mean = 0.5057 (±0.08574 SEM) (Heverin et al., 2015). The canonical receptor for 27-OH, LxRβ, was not significantly changed between controls and 27-OH treatments (Figure 5F); Vehicle (*n* = 6), mean = 1.000 (±0.1521 SEM); 27-OH (*n* = 5), mean = 2.209 (±0.6330 SEM); however, we detected a downregulation in RxRγ in the 3D co-cultures at 3DIV (Figure 5G), when the levels of this transcript should be high to promote normal neuronal differentiation. Vehicle: mean = 1 (±0.1962 SEM); 27-OH mean = 0.5600, (±0.1532 SEM), *p* = 0.0357.

## Discussion

Our findings from this study reveal a possible mechanism linking oxysterol imbalance in the brain, particularly the elevated levels of 27-hydroxycholesterol (27-OH), to synaptic dysfunction through its effects on astrocyte glutamate recapture. We used a combination of *in vivo* experiments in Cyp27Tg mice, which exhibit brain cholesterol imbalance, and an innovative 3D co-culture system that mimics the complex cellular interactions in the brain. We found that elevated 27-OH levels detrimentally affect astrocyte function *in vivo* and *in vitro*. In the CYp27Tg context, excess of 27-OH comes from different tissues in the periphery, including the liver and skeletal muscle (Meir et al., 2002), and enters the brain with several detrimental effects on neurons that we previously reported (Merino-Serrais et al., 2019; Loera-Valencia et al., 2021a,b; Goikolea et al., 2023). Similar effects are expected from chronic hypercholesterolemia induced by HFD chronically (Mateos et al., 2009). However, little information was available about the effect of excess 27-OH in astrocytes and other glial cell types, other than the induction of sterile inflammation and a reactive phenotype (Meir et al., 2002; Lodeiro et al., 2017; Loera-Valencia et al., 2021a), but information on the effects on astrocytic glutamate function was unknown.

Our study provides evidence for the downregulation of glutamate transporters GLT-1 and GLAST in astrocytes exposed to elevated 27-OH levels. These results are similar to the observed GLT-1 downregulation and neuronal hyperexcitability of AD (Masliah et al., 1996; Rothstein et al., 1996; Kirvell et al., 2006; Jacob et al., 2007; Palop et al., 2007; Bechtholt-Gompf et al., 2010; Mookherjee et al., 2011; Scott et al., 2011; Takahashi et al., 2015). The role of astrocytes in synaptic function, particularly in glutamate recapture, is crucial for maintaining cognitive performance and behavior (Garcia-Esparcia et al., 2018), and our study provides information on a specific mechanism through which 27-OH contributes to synaptic dysregulation from hypercholesterolemia, a non-genetic risk factor for AD.

Our 3D co-culture system, which allows for the differentiation of all cell types from mouse embryos, recapitulated all the effects observed in 2D neuron and 2D astrocyte cultures and the brains of Cyp27Tg mice. These results suggest a potential link between astrocytic function and synaptic dysfunction observed in the CYP27Tg model (Maioli et al., 2012; Ismail et al., 2017; Merino-Serrais et al., 2019; Loera-Valencia et al., 2021b) since our electrophysiological recordings show that high 27-OH levels induced increased overall excitability in the 3D co-cultured neurons through astrocytes, which can lead to glutamate toxicity. This mechanism would be present in AD patients with a history of hypercholesterolemia; however, a model to study these two factors in combination does not exist to the best of our knowledge. We propose that the downregulation of GLT-1 and GLAST due to high levels of 27-OH plays an important role in this aberrant phenotype. The impact of 27-OH on astrocytes, including their activation and induction of sterile inflammation, aligns with the stress signals identified for both neurons and astrocytes by our group (Lodeiro et al., 2017; Loera-Valencia et al., 2021a) and others (Staurenghi et al., 2021).

We identified novel degenerative effects in astrocytes in the 3D co-culture system that were not apparent in traditional 2D primary cultures. This emphasizes the importance of studying cellular interactions in a three-dimensional context to better mimic the brain’s cellular and physical microenvironment (Pasca, 2018). Comparing the effects of 2D primary astrocytes alone with the phenotype found in the 3D co-cultures, we can hypothesize that the effects of 27-OH in the brain are not cell-specific, and the interaction between several cell types could generate the detrimental effects observed in neurons and astrocytes and, as a limitation to this study, those interactions are present in our 3D cultures but not characterized. Previous study from our laboratory suggests that 27-OH might influence oligodendrocyte maturation (Alanko et al., 2023), and single-cell sequencing data show key enzymes for oxysterol metabolism expressed in different brain cell types (Zeisel et al., 2015; Swartzlander et al., 2018), but functional studies are yet to be performed to unravel the interaction between other cells types than neurons and astrocytes under elevated 27-OH levels.

The downregulation of glutamate transporters and the resulting hyperexcitability observed in our 3D co-culture system indicate a potential cascade effect leading to synaptic dysfunction. We did not find significant changes in LxRβ, a canonical receptor for 27-OH in the brain; however, we previously suggested for neurons that the effector cascade involved dimerization with RxRγ (Merino-Serrais et al., 2019). While we previously found that RxRγ mediated RAGE increase in 2D primary astrocytes from 27-OH (Loera-Valencia et al., 2021a), our 3D co-cultures found similar levels between controls and 27-OH treatments after 10DIV. Nevertheless, there was a significant difference in the cultures at 3DIV, when RxRγ is crucial for neuronal differentiation (Mounier et al., 2015; Simandi et al., 2018). Similarly, RxRγ induces the transcription factor REST in 2D primary cultures treated with 27-OH (Merino-Serrais et al., 2019), but mRNA levels were like controls in our 3D co-cultures, and the dendrite growth of its neurons was indeed compromised, which suggests that early 27-OH induced alterations in RxRγ signaling are sufficient to induce neuronal and astrocytic effects observed in this study. Our results also suggest a potential link between astrocytic function and the cognitive deficits observed in AD since the impact of 27-OH on astrocytes, including their activation and induction of sterile inflammation, aligns with the stress signals identified for both neurons and astrocytes (Lodeiro et al., 2017; Loera-Valencia et al., 2021a; Staurenghi et al., 2021). These findings imply a broader role for oxysterols such as 27-OH in disrupting astrocyte synaptic function, with implications for cognitive function and neurodegeneration.

## Data availability statement

The raw data supporting the conclusions of this article will be made available by the authors, without undue reservation.

## Ethics statement

The animal study was approved by Swedish Board of Agriculture (ethical permits ID S33-13, extension 57-15 and 4884/2019). The study was conducted in accordance with the local legislation and institutional requirements.

## Author contributions

FS: Data curation, Formal analysis, Investigation, Methodology, Software, Visualization, Writing – review & editing. GG: Conceptualization, Investigation, Methodology, Writing – review & editing. JG: Data curation, Formal analysis, Investigation, Writing – review & editing. ML-L: Data curation, Formal analysis, Investigation, Writing – review & editing. HB-T: Data curation, Formal analysis, Investigation, Methodology, Software, Visualization, Writing – review & editing. KG: Writing – review & editing, Investigation, Methodology. LÁ-J: Writing – review & editing, Investigation, Methodology. AP: Writing – review & editing, Conceptualization, Investigation, Methodology, Supervision, Visualization. MG-G: Writing – review & editing, Conceptualization, Investigation, Methodology, Supervision. AF: Writing – review & editing, Resources. AC-M: Funding acquisition, Investigation, Resources, Supervision, Writing – review & editing. SM: Funding acquisition, Investigation, Project administration, Resources, Supervision, Writing – review & editing. RL-V: Conceptualization, Data curation, Formal analysis, Funding acquisition, Investigation, Methodology, Project administration, Resources, Software, Supervision, Validation, Visualization, Writing – original draft, Writing – review & editing.
