# Supplementary material for: Impaired astrocytic synaptic function by peripheral cholesterol metabolite 27-hydroxycholesterol
Source: Front Cell Neurosci. 2024 Apr 8;18:1347535. doi: 10.3389/fncel.2024.1347535 (PMC11034371; doi:10.3389/fncel.2024.1347535)
Supplement: Supplementary file 1 [file Image_1.pdf]

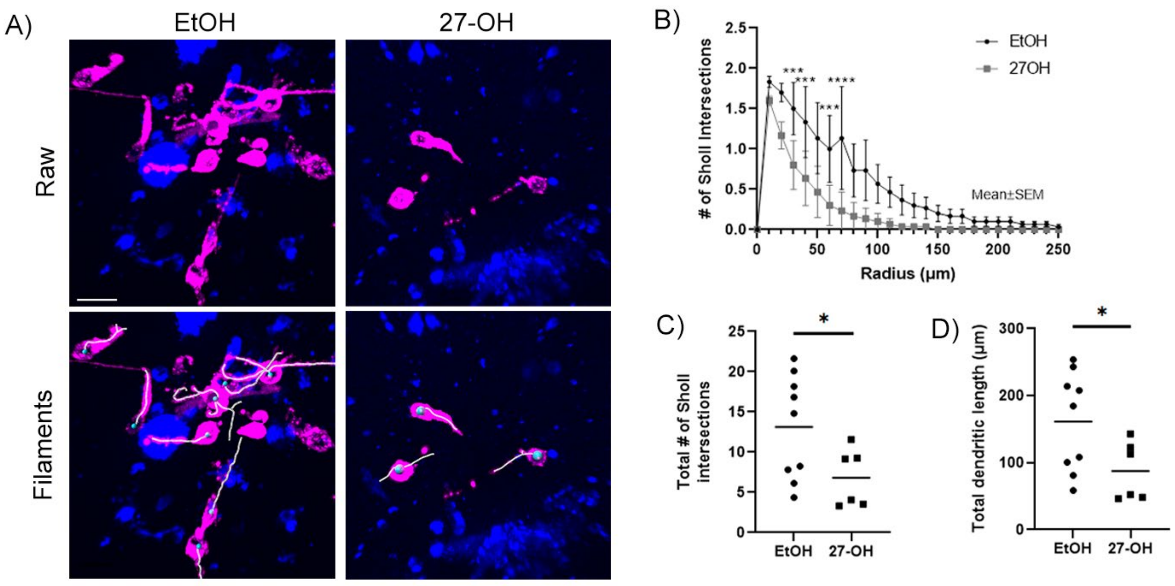

Figure S1. **27-OH treatments reduce neuron complexity in 3D co-cultures**. Morphological analysis of MAP2-stained neurons (magenta). DAPI (blue) was used to stain the nuclei. A) Representative images of neurons treated with DMSO or 27-OH (top row) and rendering of neuronal filaments using Imaris software (bottom row). B-D) Quantification of the number of Sholl intersections at different radii (B), number of Sholl intersections per neuron (C) and dendritic length per neuron (D). Only neurons with at least one process were counted. Data points represent the mean of each dish imaged and the bar the total mean of each condition. Total biological replicates n=15 (*P<0.05, ***P<0.001, ****P<0.0001). Scale bar is 20 μm and applies to all images.

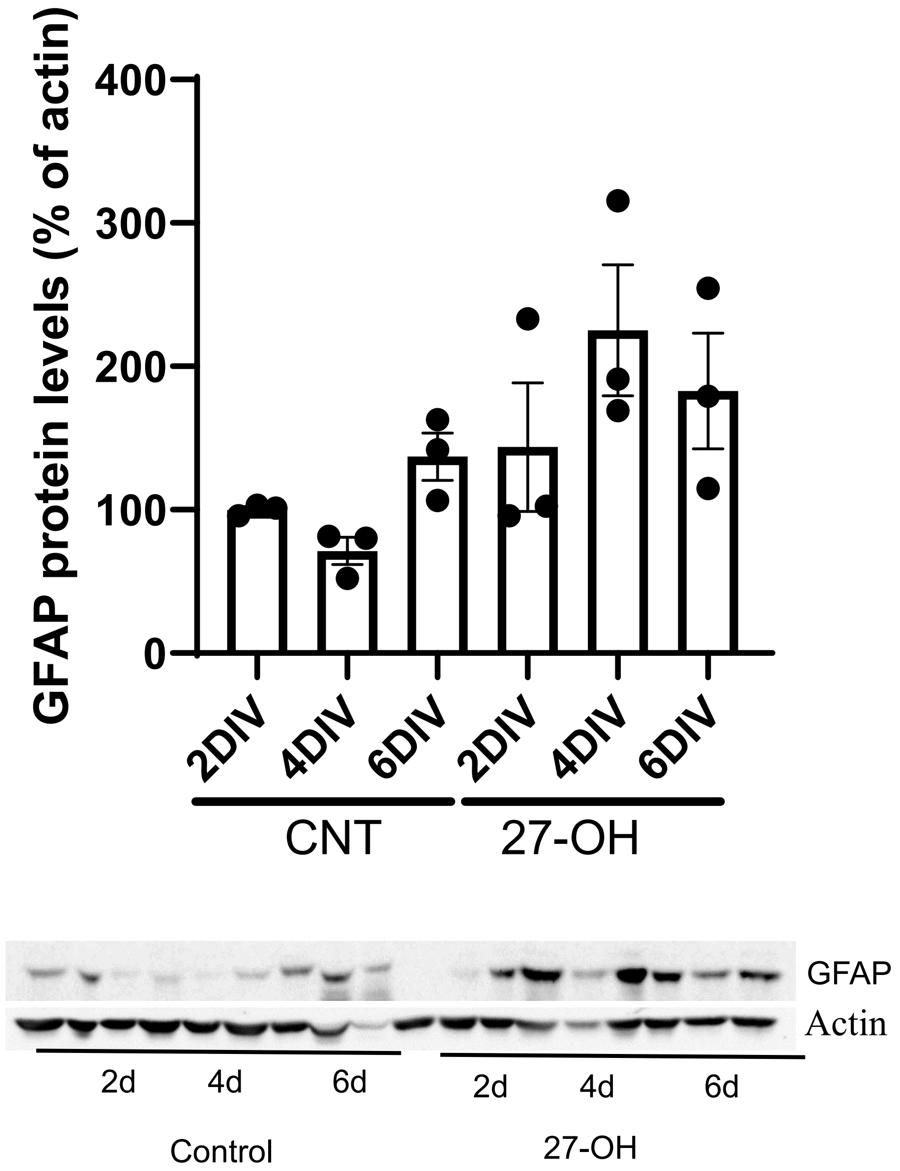

Figure S2. Densitometric analysis of western blots from 2D primary astroglial cultures treated with 1 μM of 27-OH shows increased protein levels of GFAP. Graph shows protein collected from cultures at days 2, 4 and 6 *in vitro*. Normalized to 2DIV CNT (%).
